# Supplementary material for: The Joint Influence of Tl+ and Thiol-Modifying Agents on Rat Liver Mitochondrial Parameters In Vitro
Source: Int J Mol Sci. 2022 Aug 11;23(16):8964. doi: 10.3390/ijms23168964 (PMC9409397; doi:10.3390/ijms23168964)
Supplement: Supplementary file 1 [file ijms-23-08964-s001.zip › ijms-1801181-supplementary.pdf]

Supplementary Material

Supplementary data for the manuscript:

The joint influence of Tl^+ and thiol-modifying agents on rat liver mitochondrial parameters *in vitro*

Sergey M. Korotkov and Artemy V. Novozhilov

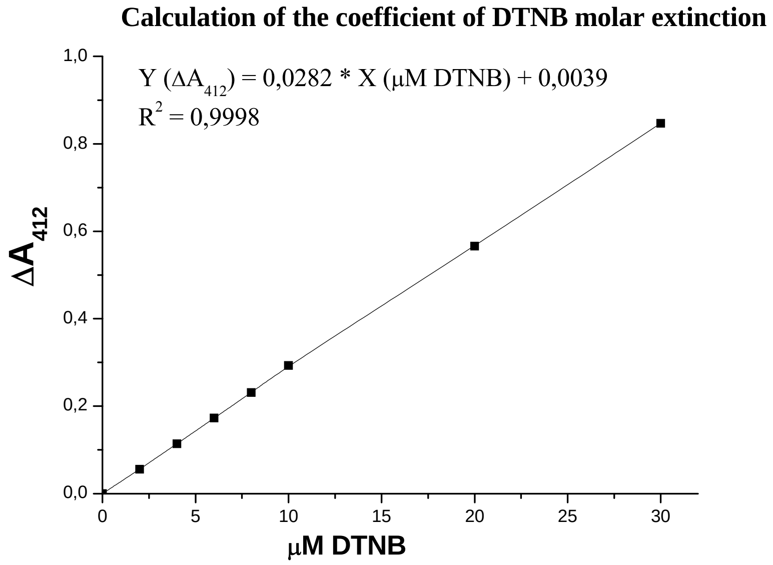

Figure S1. Calculation of the DTNB molar extinction coefficient. Tabular data for the coefficient are 14400. Titration of micromolar concentrations of DTNB was performed with dithiothreitol. In this case, two molecules of thionitrobenzoic acid are formed; therefore, the calculated coefficient was divided by two. Formula for calculating the coefficient: 0.0282 * 1000000/2 = 14100 (97,9%).

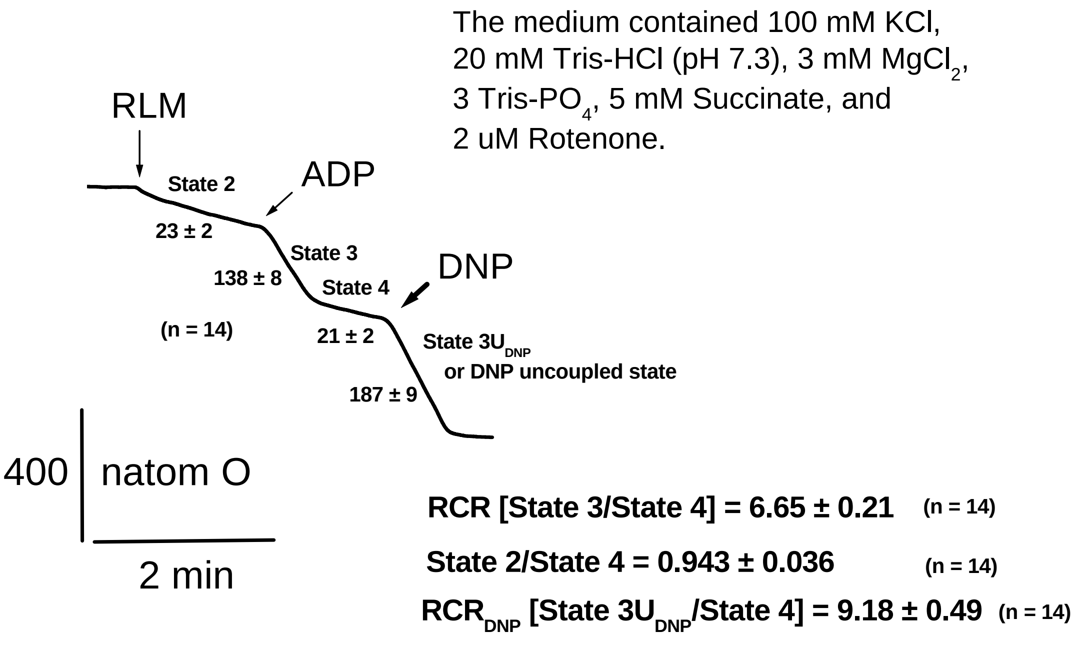

Typical oxygraphs and RCR for control RLM experiments *in vitro*

Figure S2. Typical oxygraph and RCR to control rat liver mitochondrial preparations.

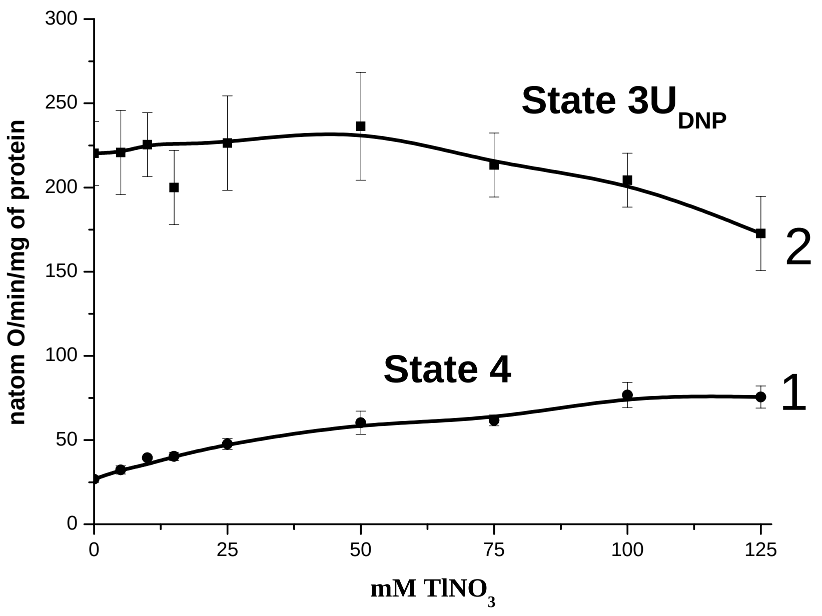

(see Korotkov et al., 2008 more detail)

Figure S3. Effect of Tl^+ on oxygen consumption rates (natom O min/mg of protein) in succinate-energized rat liver mitochondria. Mitochondria (1.5 mg/mL of protein) were injected in sucrose-adjusted 280 mOsm medium containing 0-125 mM $TlNO_3$ (traces 1 and 2) and 5 mM Tris-NO_3 (pH 7.3), 5 mM succinate, 4 μM rotenone, 3 mM $Mg(NO_3)_2$, and 3 mM Tris-P_i. 2,4-dinitrophenol (DNP) of 30 μM was administered into the medium to trigger DNP-stimulated respiration (trace 2) after 2 min recording of state 4 (trace 1). Error bars were calculated by the Muller formula from rates found for three different mitochondrial preparations.

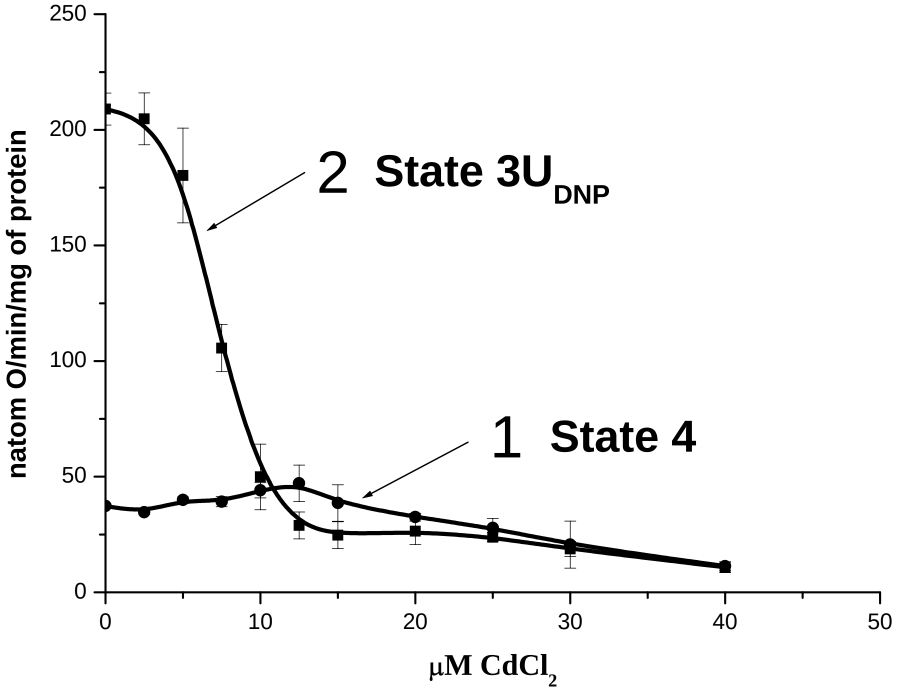

(see Korotkov et al., 1998 more detail)

Figure S4. Effect of Cd^{2+} on oxygen consumption rates (natom O min/mg of protein) in succinate-energized rat liver mitochondria. Mitochondria (2 mg/mL of protein) were injected in 280 mOsm medium containing 100 mM KCl (traces 1 and 2), 20 mM Tris-HCl (pH 7.3), 5 mM succinate, 4 μM rotenone, 3 mM $MgCl_2$, and 3 mM Tris-P_i . 2,4-dinitrophenol (DNP) of 30 μM was administered into the medium to trigger DNP-stimulated respiration (trace 2) after 2 min recording of state 4 (trace 1). Error bars were calculated by the Muller formula from rates found for three different mitochondrial preparations.

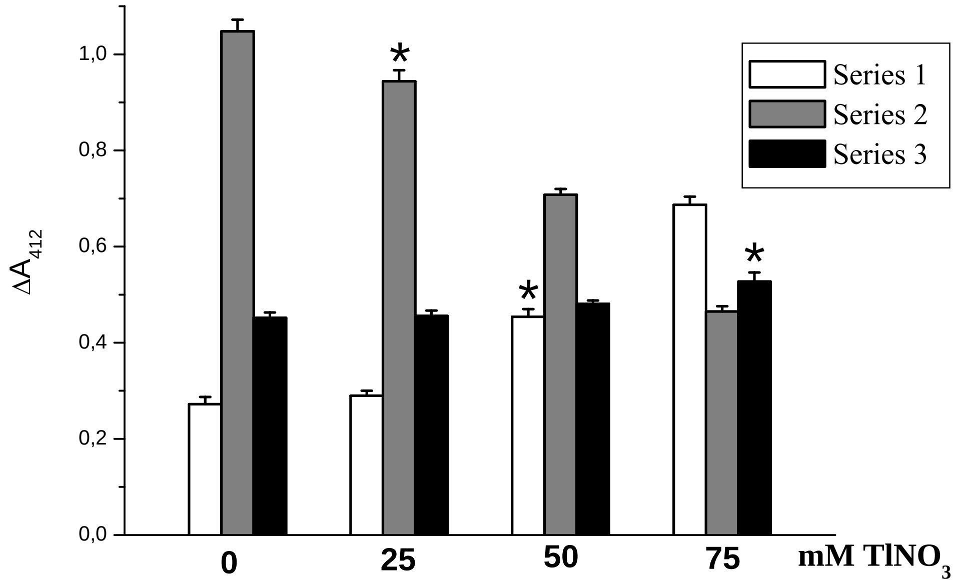

(see Korotkov et al., 2014 more detail)

Figure S5. Effects of Tl^{+} on protein thiol content of rat liver mitochondria in medium with $TlNO_3$ and KNO_3. Mitochondria (1 mg protein/mL) were supplemented to sucrose-adjusted 400 mOsm medium containing 0–75 mM $TlNO_3$, 125 mM KNO_3, 5 mM Tris-NO_3 (pH 7.3), 5 mM succinate, 2 µM rotenone, and 1 µg/mL of oligomycin. Triple freezing after 4 min mitochondrial incubation in the above medium was carried out in the medium containing $TlNO_3$ and KNO_3 (Series 1 and 2) or in a medium (series 3) containing 125 mM KNO_3 and 5 mM Tris-NO_3 (pH 7.3). Data for the inner membrane fraction are shown in series of 1 or 3. Data for the matrix soluble protein fraction are shown in series of 2. Error bars were calculated with Muller's formula from duplicate magnitudes of ΔA_{412} found for four different mitochondrial preparations. Asterisks indicate statistically significant deviation of the content mean for a thallium-free medium.

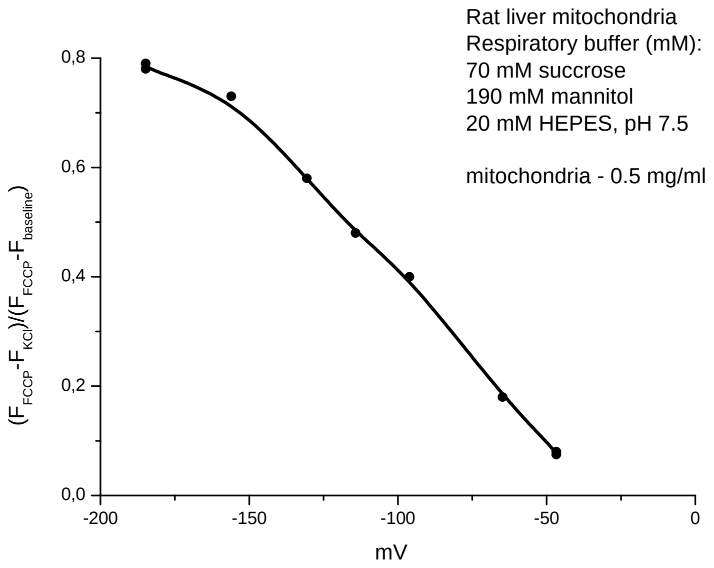

Figure S6. Calibration in the dependence of safranin fluorescence on ΔΨ. The safranin fluorescence was evaluated at 20 °C using a Shimadzu RF-1501 spectrofluorimeter (Shimadzu, Japan) at 485/590 nm wavelength (excitation/emission). To record $F_{baseline}$ (2 min), mitochondria (0.5 mg/ml of protein) were injected into a quartz transparent cuvette filled by 3 ml of the medium containing 190 mM mannitol, 70 mM sucrose, 5 μM rotenone, 2.5 μM safranin, 20 nM valinomycin, 4 μg/ml of oligomycin, and 20 mM HEPES, brought to pH 7.5 with 1 M NaOH. To register F_{KCl} (2 min), concentrated KCl solution was farther injected into the cuvette to reach final concentrations of 0.1, 0.3, 0.8, 1.5, 3, 10, and 20 mM K^+. Finally, 1 μM FCCP was added into the cuvette with subsequent 2 min registration of F_{FCCP}. From these data, $(F_{FCCP} - F_{KCl})/(F_{FCCP} - F_{baseline})$ was calculated for each measurement and plotted against ΔΨ, calculated using the Nernst equation.

Table S1

Effects of EMA, FITC, embeline, and $Cu(OP)_2$ on the absorbance change (ΔA_{540}) in the suspension of succinate-energized rat liver mitochondria

**EMA**			**FITC**			**$Cu(OP)_2$**		
**EMA** (µM)	$\Delta A_{540} \pm$ SEM	P value	**FITC** (µM)	$\Delta A_{540} \pm$ SEM	P value	**$Cu(OP)_2$** (µM)	$\Delta A_{540} \pm$ SEM	P value
0	-0.025±0.003 (4)	-	0	-0.025±0.002 (6)	-	0	-0.025±0.003 (4)	-
10	-0.019±0.003 (3)	*	30	-0.004±0.006 (5)	$P < 0.01$	3	-0.034±0.003 (4)	*
30	0.002±0.006 (3)	$P < 0.01$	100	0.055±0.006 (5)	$P < 0.01$	3 + ADP + CsA + NEM	-0.080±0.007 (3)	$P < 0.01$
50	-0.011±0.002 (3)	$P < 0.01$	300	-0.018±0.004 (4)	*	3 + CsA + NEM	-0.121±0.002 (3)	$P < 0.01$
						3 + ADP + NEM	-0.149±0.005 (3)	$P < 0.01$
						3 + NEM	-0.179±0.008 (3)	$P < 0.01$

**Embeline (Emb)**		
**Emb** (µM)	$\Delta A_{540} \pm$ SEM	P value
0	-0.008±0.002 (7)	-
50	-0.063±0.007 (6)	$P < 0.01$
100	-0.086±0.005 (6)	$P < 0.01$

The changes in ΔA_{540} were detected within six minute interval after the addition of EMA, FITC, Emb, or $Cu(OP)_2$ (see Fig. 1) and presented as Means ± SEM. The number of experiments showed in parentheses. P-values were accordingly calculated to experiments free of MSL (a dash in the P value columns). Asterisks indicate that statistical difference between appropriate ΔA_{540} values is not statistically significant.

Table S2 (Part 1)

Effects of EMA and FITC on the absorbance change (ΔA_{540}) the suspension of non-energized rat liver mitochondria

**EMA** (μM)	**EMA**				**FITC** (μM)	**FITC**			
	- ADP		+ ADP			- ADP		+ ADP	
	ΔA_{540} ± SEM	P value	ΔA_{540} ± SEM	P value		ΔA_{540} ± SEM	P value	ΔA_{540} ± SEM	P value
0	-0.105±0.008 (3)	-		-	0	-0.105±0.008 (3)	-		-
5	-0.096±0.005 (3)	*	-0.099±0.004 (3)	*	30	-0.102±0.009 (3)	*	-0.105±0.005 (3)	*
10	-0.084±0.010 (3)	*	-0.083±0.005 (3)	P < 0.02	100	-0.135±0.004 (3)	P < 0.01	-0.120±0.004 (3)	P < 0.03
30	-0.036±0.034 (3)	*	-0.070±0.008 (3)	P < 0.02	200	-0.210±0.007 (3)	P < 0.01	-0.157±0.011 (3)	P < 0.02
50	0.022±0.012 (3)	P < 0.01	-0.013±0.008 (3)	P < 0.01	300	-0.256±0.011 (3)	P < 0.01	-0.214±0.015 (3)	P < 0.01

The changes in ΔA_{540} (see Fig. 2) were detected within three minute interval after addition of mitochondria into the medium containing 75 mM $TlNO_3$ and 125 mM KNO_3 and presented as Means ± SEM. The number of experiments showed in parentheses. P-values were accordingly calculated to experiments free of EMA or FITC (a dash in the P value columns). Asterisks indicate that statistical difference between appropriate ΔA_{540} values is not statistically significant.

Table S2 (Part 2)

Effects of EMA and FITC on the absorbance change (ΔA_{540}) in the suspension of succinate-energized rat liver mitochondria

**EMA** (μM)	**EMA**				**FITC** (μM)	**FITC**			
	- ADP		+ ADP			- ADP		+ ADP	
	ΔA_{540} ± SEM	P value	ΔA_{540} ± SEM	P value		ΔA_{540} ± SEM	P value	ΔA_{540} ± SEM	P value
0	0.087±0.005 (3)	-		-	0	0.087±0.005 (3)	-		-
5	0.089±0.017 (3)	*	0.096±0.005 (3)	*	30	0.093±0.016 (3)	*	0.099±0.008 (3)	*
10	0.080±0.014 (3)	*	0.070±0.004 (3)	P < 0.04	100	0.202±0.015 (3)	P < 0.01	0.164±0.009 (3)	P < 0.01
30	0.049±0.021 (3)	*	0.052±0.003 (3)	P < 0.01	200	0.209±0.021 (3)	P < 0.01	0.217±0.012 (3)	P < 0.01
50	0.075±0.005 (3)	*	0.094±0.005 (3)	*	300	0.197±0.025 (3)	P < 0.01	0.183±0.016 (3)	P < 0.01

The changes in ΔA_{540} (see Fig. 2) were detected within four minute interval after addition of 5 mM succinate into the medium containing 75 mM $TlNO_3$ and 125 mM KNO_3 and presented as Means ± SEM. The number of experiments showed in parentheses. P-values were accordingly calculated to experiments free of EMA or FITC (a dash in the P value columns). Asterisks indicate that statistical difference between appropriate ΔA_{540} values is not statistically significant.

Table S3 (Part 1)

Effects of Ca^{2+}, EMA and FITC on the absorbance change (ΔA_{540}) in the suspension of non-energized rat liver mitochondria

EMA (μM)	EMA				FITC (μM)	FITC			
	- Ca^{2+}		+ Ca^{2+}			- Ca^{2+}		+ Ca^{2+}	
	ΔA_{540} ± SEM	P value	ΔA_{540} ± SEM	P value		ΔA_{540} ± SEM	P value	ΔA_{540} ± SEM	P value
0	-0.105±0.008 (3)	-	-0.093±0.005 (3)	-	0	-0.105±0.008 (3)	-	-0.093±0.005 (3)	-
5	-0.096±0.005 (3)	*	-0.079±0.004 (3)	*	30	-0.102±0.009 (3)	*	-0.105±0.005 (3)	*
10	-0.084±0.010 (3)	*	-0.068±0.009 (3)	*	100	-0.135±0.004 (3)	P < 0.01	-0.120±0.004 (3)	P < 0.03
30	-0.036±0.034 (3)	*	-0.039±0.030 (3)	*	200	-0.210±0.007 (3)	P < 0.01	-0.157±0.011 (3)	P < 0.02
50	0.022±0.012 (3)	P < 0.01	-0.007±0.029 (3)	P < 0.03	300	-0.256±0.011 (3)	P < 0.01	-0.214±0.015 (3)	P < 0.01

The changes in ΔA_{540} (see Fig. 3) were detected within three minute interval after addition of mitochondria into the medium containing 75 mM $TlNO_3$ and 125 mM KNO_3 and presented as Means ± SEM. The number of experiments showed in parentheses. P-values were accordingly calculated to experiments with EMA or FITC, and without/with 50 μM Ca^{2+} (a dash in the P value columns). Asterisks indicate that statistical difference between appropriate ΔA_{540} values is not statistically significant.

Table S3 (Part 2)

Effects of Ca^{2+}, EMA and FITC on the absorbance change (ΔA_{540}) in the suspension of succinate-energized rat liver mitochondria

**EMA** (μM)	**EMA**				**FITC** (μM)	**FITC**			
	- Ca^{2+}		+ Ca^{2+}			- Ca^{2+}		+ Ca^{2+}	
	ΔA_{540} ± SEM	P value	ΔA_{540} ± SEM	P value		ΔA_{540} ± SEM	P value	ΔA_{540} ± SEM	P value
0	0.087±0.005 (3)	-	-0.135±0.007 (3)	-	0	0.087±0.005 (3)	-	-0.135±0.007 (3)	-
5	0.089±0.017 (3)	*	-0.195±0.015 (3)	P < 0.03	30	0.093±0.016 (3)	*	-0.165±0.009 (3)	*
10	0.080±0.013 (3)	*	-0.222±0.011 (3)	P < 0.01	100	0.202±0.015 (3)	P < 0.01	-0.160±0.017 (3)	*
30	0.049±0.020 (3)	*	-0.199±0.034 (3)	P < 0.01	200	0.209±0.021 (3)	P < 0.01	0.085±0.023 (3)	P < 0.01
50	0.075±0.005 (3)	*	-0.200±0.014 (3)	P < 0.01	300	0.197±0.025 (3)	P < 0.01	0.125±0.014 (3)	P < 0.01

The changes in ΔA_{540} (see Fig. 3) were detected within four minute interval after addition of 5 mM succinate into the medium containing 75 mM $TlNO_3$ and 125 mM KNO_3 and presented as Means ± SEM. The number of experiments showed in parentheses. P-values were accordingly calculated to experiments with EMA or FITC, and without/with 50 μM Ca^{2+} (a dash in the P value columns). Asterisks indicate that statistical difference between appropriate ΔA_{540} values is not statistically significant.

Table 3S (Part 3)

Effects of TFP on the absorbance change (ΔA_{540}) in the suspension of rat liver mitochondria

TFP (μM)	non-energized RLM (a)				TFP (μM)	succinate-energized RLM (b)			
	- Ca^{2+}		+ Ca^{2+}			- Ca^{2+}		+ Ca^{2+}	
	ΔA_{540} ± SEM	P value	ΔA_{540} ± SEM	P value		ΔA_{540} ± SEM	P value	ΔA_{540} ± SEM	P value
0	-0.109±0.004 (3)	-	-0.107±0.004 (3)	-	0	0.092±0.005 (3)	-	-0.056±0.013 (3)	-
15	-0.125±0.003 (3)	P < 0.05	-0.119±0.005 (3)	*	15	0.114±0.006 (3)	P < 0.05	-0.082±0.027 (3)	*
25	-0.125±0.005 (3)	*	-0.130±0.009 (3)	*	25	0.115±0.002 (3)	P < 0.02	-0.012±0.033 (3)	*
50	-0.131±0.006 (3)	P < 0.04	-0.139±0.008 (3)	P < 0.03	50	0.062±0.006 (3)	P < 0.02	-0.019±0.033 (3)	*
100	-0.156±0.011 (3)	P < 0.02	-0.153±0.010 (3)	P < 0.03	100	-0.009±0.001 (3)	P < 0.01	-0.015±0.003 (4)	P < 0.02

The changes in ΔA_{540} (see Fig. 3) were detected within three (a) or four (b) minute interval accordingly after addition of mitochondria (a) or 5 mM succinate (b) into the medium containing 75 mM $TlNO_3$ and 125 mM KNO_3 and presented as Means ± SEM. The number of experiments showed in parentheses. P-values were accordingly calculated to experiments with TFP and without/with 50 μM Ca^{2+} (a dash in the P value columns). Asterisks indicate that statistical difference between appropriate ΔA_{540} values is not statistically significant.

Table S4

Effects of EMA, FITC, $Cu(OP)_2$, and Emb on the absorbance change (ΔA_{540}) in the suspension of succinate-energized and calcium-loaded rat liver mitochondria

**EMA** (μM)	**EMA**				**FITC** (μM)	**FITC**			
	Ca^{2+}		Ca^{2+} + ADP			Ca^{2+}		Ca^{2+} + ADP	
	ΔA_{540} ± SEM	P value	ΔA_{540} ± SEM	P value		ΔA_{540} ± SEM	P value	ΔA_{540} ± SEM	P value
**free of Ca^{2+}**	-0.043±0.002 (3)	P < 0.01			**free of Ca^{2+}**	-0.044±0.001 (4)	P < 0.01		
0	-0.229±0.016 (3)	-	-0.042±0.004 (3)	P < 0.01	0	-0.234±0.013 (4)	-	0.042±0.003 (4)	P < 0.01
5	-0.301±0.010 (3)	P < 0.02	-0.124±0.007 (3)	P < 0.01	30	-0.216±0.009 (4)	*	-0.054±0.012 (4)	*
10	-0.267±0.009 (3)	*	-0.155±0.005 (3)	P < 0.02	50	-0.237±0.011 (4)	*	-0.053±0.006 (4)	P < 0.01
30	-0.230±0.013 (3)	*	-0.116±0.011 (3)	P < 0.01	100	-0.227±0.016 (4)	*	-0.060±0.008 (4)	P < 0.01
					200	-0.105±0.012 (4)	P < 0.01	-0.039±0.005 (4)	P < 0.01

**$Cu(OP)_2$** (μM)	**$Cu(OP)_2$**				**Emb** (μM)	**Emb**			
	Ca^{2+}		Ca^{2+} + ADP			Ca^{2+}		Ca^{2+} + ADP	
	$\Delta A_{540} \pm$ SEM	P value	$\Delta A_{540} \pm$ SEM	P value		$\Delta A_{540} \pm$ SEM	P value	$\Delta A_{540} \pm$ SEM	P value
**free of Ca^{2+}**	-0.029±0.003 (3)	P < 0.01			0	-0.009±0.002 (7)	P < 0.01		
0	-0.212±0.010 (3)	-	-0.033±0.002 (3)	P < 0.01	0	-0.187±0.006 (7)	-	-0.009±0.001 (7)	P < 0.01
3	-0.255±0.014 (3)	P < 0.03	-0.035±0.004 (3)	P < 0.01	25	-0.173±0.006 (4)	*	-0.030±0.013 (4)	P < 0.01
					50	-0.162±0.012 (5)	*	-0.052±0.015 (5)	P < 0.01
					100	-0.181±0.015 (4)	*	-0.166±0.017 (4)	*

The change in ΔA_{540} (see Fig. 4) were detected within seven minute interval after addition of 50 μM Ca^{2+} into the medium containing 75 mM $TlNO_3$ and 125 mM KNO_3 and presented as Means ± SEM. The change was three minutes after addition of 50 μM Ca^{2+} in experiments with embeline. The number of experiments showed in parentheses. P-values were accordingly calculated to experiments with 50 μM Ca^{2+} (a dash in the P value columns). Asterisks indicate that statistical difference between appropriate ΔA_{540} values is not statistically significant.

Table S5 (Part 1)

Effects of Ca^{2+}, EMA and FITC on the absorbance change (ΔA_{540}) in the suspension of non-energized rat liver mitochondria

EMA (μM)	EMA				FITC (μM)	FITC			
	Ca^{2+}		Ca^{2+}+ ADP			Ca^{2+}		Ca^{2+}+ ADP	
	ΔA_{540} ± SEM	P value	ΔA_{540} ± SEM	P value		ΔA_{540} ± SEM	P value	ΔA_{540} ± SEM	P value
free of Ca^{2+}	-0.105±0.008 (3)	*			free of Ca^{2+}	-0.105±0.008 (3)	-		
0	-0.093±0.005 (3)	-	-0.109±0.009		0	-0.093±0.005 (3)	-	-0.109±0.009 (3)	-
5	-0.079±0.004 (3)	P < 0.01	-0.094±0.005 (3)	*	30	-0.105±0.005 (3)	*	-0.103±0.005 (3)	*
10	-0.068±0.009 (3)	P < 0.01	-0.082±0.006 (3)	P < 0.02	100	-0.120±0.004 (3)	P < 0.01	-0.115±0.008 (3)	P < 0.03
30	-0.039±0.030 (3)	P < 0.01	-0.065±0.005 (3)	P < 0.01	200	-0.157±0.011 (3)	P < 0.01	-0.166±0.005 (3)	P < 0.02
50	-0.007±0.029 (3)	P < 0.01	-0.019±0.003 (3)	P < 0.01	300	-0.214±0.015 (3)	P < 0.01	-0.164±0.008 (3)	P < 0.01

The changes in ΔA_{540} (see Fig. 5) were detected within three minute interval after addition of mitochondria into the medium containing 75 mM $TlNO_3$ and 125 mM KNO_3 and presented as Means ± SEM. The number of experiments showed in parentheses. P-values were accordingly calculated to experiments with EMA or FITC, and without/with 50 μM Ca^{2+} (a dash in the P value columns). Asterisks indicate that statistical difference between appropriate ΔA_{540} values is not statistically significant.

Table S5 (Part 2)

Effects of Ca^{2+}, EMA and FITC on the absorbance change (ΔA_{540}) in the suspension of succinate-energized rat liver mitochondria

EMA (μM)	EMA				FITC (μM)	FITC			
	Ca^{2+}		Ca^{2+}+ ADP			Ca^{2+}		Ca^{2+}+ ADP	
	ΔA_{540} ± SEM	P value	ΔA_{540} ± SEM	P value		ΔA_{540} ± SEM	P value	ΔA_{540} ± SEM	P value
free of Ca^{2+}	0.087±0.005 (3)	-			free of Ca^{2+}	0.087±0.005 (3)	-		
0	-0.135±0.007 (3)	-	0.092±0.005 (3)	P < 0.01	0	-0.135±0.007 (3)	-	0.092±0.005 (3)	-
5	-0.195±0.015 (3)	P < 0.03	0.013±.003 (3)	P < 0.01	30	-0.165±0.009 (3)	*	-0.011±.032 (3)	P < 0.01
10	-0.222±0.011 (3)	P < 0.01	0.026±0.015 (3)	P < 0.01	100	-0.160±0.017 (3)	*	0.127±0.009 (3)	P < 0.01
30	-0.199±0.034 (3)	P < 0.01	-0.009±0.007 (3)	P < 0.01	200	0.085±0.023 (3)	P < 0.01	0.186±0.019 (3)	P < 0.01
50	-0.200±0.014 (3)	P < 0.01	0.019±0.003 (3)	P < 0.01	300	0.125±0.014 (3)	P < 0.01	0.165±0.014 (3)	P < 0.01

The changes in ΔA_{540} (see Fig. 5) were detected within four minute interval after addition of 5 mM succinate into the medium containing 75 mM $TlNO_3$ and 125 mM KNO_3 and presented as Means ± SEM. The number of experiments showed in parentheses. P-values were accordingly calculated to experiments with EMA or FITC, and without/with 50 μM Ca^{2+} (a dash in the P value columns). Asterisks indicate that statistical difference between appropriate ΔA_{540} values is not statistically significant.

Table S6

Effects of EMA, FITC, $Cu(OP)_2$, and Emb on the absorbance change (ΔA_{540}) in the suspension of succinate-energized and calcium-loaded rat liver mitochondria in the presence of MPTP inhibitors (ADP, CsA, NEM)

EMA				FITC			
	**EMA** (μM)	ΔA_{540} ± SEM	P value		**FITC** (μM)	ΔA_{540} ± SEM	P value
free of Ca^{2+}	0	-0.030±0.007 (4)	P < 0.01	free of Ca^{2+}	0	-0.038±0.005 (4)	P < 0.01
Ca^{2+}	0	-0.216±0.013 (4)	-	Ca^{2+}	0	-0.227±0.012 (4)	-
Ca^{2+}	10	-0.252±0.009 (4)	*	Ca^{2+}	100	-0.233±0.014 (4)	*
Ca^{2+} + ADP	10	-0.139±0.008 (4)	P < 0.01	Ca^{2+} + ADP	100	-0.066±0.014 (4)	P < 0.01
Ca^{2+} + CsA	10	-0.177±0.005 (4)	P < 0.05	Ca^{2+} + CsA	100	-0.145±0.007 (3)	P < 0.01
Ca^{2+} + NEM	10	-0.171±0.006 (4)	P < 0.03	Ca^{2+} + NEM	100	-0.166±0.008 (3)	P < 0.02
Ca^{2+} + ADP + CsA	10	-0.120±0.008 (3)	P < 0.01	Ca^{2+} + ADP + CsA	100	-0.141±0.006 (3)	P < 0.01
Ca^{2+} + ADP + NEM	10	-0.099±0.011 (3)	P < 0.01	Ca^{2+} + ADP + NEM	100	-0.134±0.008 (3)	P < 0.01
Ca^{2+} + CsA + NEM	10	-0.173±0.011 (4)	P < 0.05	Ca^{2+} + CsA + NEM	100	-0.177±0.006 (3)	P < 0.02

Cu(OP)$_2$				Emb			
	**Cu(OP)$_2$** (μM)	ΔA_{540} ± SEM	P value		**Emb** (μM)	ΔA_{540} ± SEM	P value
free of Ca^{2+}	0	-0.018±0.002 (3)	P < 0.01	free of Ca^{2+}	0	-0.010±0.001 (8)	P < 0.01
Ca^{2+}	0	-0.214±0.006 (3)	-	Ca^{2+}	0	-0.189±0.005 (8)	-
Ca^{2+} + ADP	0	-0.025±0.006 (3)	P < 0.01	Ca^{2+} + ADP	0	-0.009±0.001 (8)	P < 0.01
Ca^{2+}	3	-0.230±0.011 (3)	*	Ca^{2+}	50	-0.162±0.012 (6)	*
Ca^{2+} + ADP	3	-0.015±0.002 (3)	P < 0.01	Ca^{2+} + ADP	50	-0.054±0.012 (6)	P < 0.01
Ca^{2+} + CsA	3	-0.011±0.001 (3)	P < 0.01	Ca^{2+} + CsA	50	-0.190±0.005 (3)	*
Ca^{2+} + NEM	3	-0.288±0.014 (3)	P < 0.01	Ca^{2+} + NEM	50	-0.121±0.010 (3)	P < 0.01
Ca^{2+} + ADP + NEM	3	-0.057±0.007 (3)	P < 0.01	Ca^{2+} + ADP + CsA	50	-0.027±0.003 (3)	P < 0.01
Ca^{2+} + CsA + NEM	3	-0.282±0.012 (3)	P < 0.01	Ca^{2+} + ADP + NEM	50	-0.017±0.002 (3)	P < 0.01
Ca^{2+} + ADP + CsA + NEM	3	-0.018±0.005 (3)	P < 0.01	Ca^{2+} + CsA + NEM	50	-0.059±0.004 (3)	P < 0.01

The change in ΔA_{540} (see Fig. 6) was detected within the minute interval of three for Cu(OP)$_2$ and embeline or five for EMA and FITC after addition of 50 μM Ca^{2+} into the medium containing 75 mM $TlNO_3$ and 125 mM KNO_3 and presented as Means ± SEM. The number of experiments showed in parentheses. P-values were accordingly calculated to experiments with 50 μM Ca^{2+} (a dash in the P value columns). Asterisks indicate that statistical difference between appropriate ΔA_{540} values is not statistically significant.

Table S7

Effects of EMA, FITC, $Cu(OP)_2$, and TFP on the absorbance change (ΔA_{540}) in the suspension of succinate-energized and calcium-loaded rat liver mitochondria in the presence of MPTP inhibitors (ADP, CsA, NEM)

EMA				FITC			
	**EMA** (µM)	ΔA_{540} ± SEM	P value		**FITC** (µM)	ΔA_{540} ± SEM	P value
free of Ca^{2+}	0	0.091±0.005 (3)	P < 0.01	free of Ca^{2+}	0	0.091±0.005 (3)	P < 0.01
Ca^{2+}	0	-0.153±0.012 (3)	-	Ca^{2+}	0	-0.153±0.012 (3)	-
Ca^{2+}	10	-0.228±0.009 (3)	P < 0.01	Ca^{2+}	100	-0.160±0.017 (3)	P < 0.01
Ca^{2+} + ADP	10	0.037±0.007 (3)	P < 0.01	Ca^{2+} + ADP	100	0.127±0.009 (3)	P < 0.01
Ca^{2+} + CsA	10	0.001±0.020 (3)	P < 0.01	Ca^{2+} + CsA	100	0.159±0.010 (3)	P < 0.01
Ca^{2+} + NEM	10	0.020±0.008 (3)	P < 0.01	Ca^{2+} + NEM	100	0.143±0.011 (3)	P < 0.01
Ca^{2+} + Mg^{2+}	10	-0.095±0.005 (3)	P < 0.01	Ca^{2+} + Mg^{2+}	100	-0.073±0.009 (3)	P < 0.01

**$Cu(OP)_2$**				**TFP**			
	**$Cu(OP)_2$** (μM)	ΔA_{540} ± SEM	P value		**TFP** (μM)	ΔA_{540} ± SEM	P value
free of Ca^{2+}	0	0.067±0.010 (3)	$P < 0.01$	free of Ca^{2+}	0	0.099±0.003 (8)	$P < 0.01$
Ca^{2+}	0	-0.161±0.008 (3)	-	Ca^{2+}	0	-0.081±0.006 (8)	-
Ca^{2+}	3	-0.159±0.006 (3)	*	Ca^{2+}	50	-0.095±0.008 (6)	*
Ca^{2+} + ADP	3	-0.024±0.003 (3)	$P < 0.01$	Ca^{2+} + ADP	50	-0.146±0.012 (6)	$P < 0.01$
Ca^{2+} + CsA	3	0.039±0.017 (3)	$P < 0.01$	Ca^{2+} + CsA	50	-0.034±0.004 (3)	$P < 0.01$
Ca^{2+} + NEM	3	-0.098±0.011 (3)	$P < 0.01$	Ca^{2+} + NEM	50	-0.011±0.003 (3)	$P < 0.01$
Ca^{2+} + ADP + CsA	3	0.075±0.003 (3)	$P < 0.01$	Ca^{2+} + ADP + CsA	50	-0.019±0.002 (3)	$P < 0.01$
Ca^{2+} + ADP + NEM	3	-0.103±0.004 (3)	$P < 0.01$	Ca^{2+} + CsA + NEM	50	-0.012±0.004 (3)	$P < 0.01$
Ca^{2+} + CsA + NEM	3	-0.22±0.004 (3)	$P < 0.01$	Ca^{2+} + Mg^{2+}	50	-0.057±0.004(3)	$P < 0.01$

The change in ΔA_{540} (see Fig. 7) was detected within seven minute after succinate addition into the medium containing 75 mM $TlNO_3$ and 125 mM KNO_3 and presented as Means ± SEM. The number of experiments showed in parentheses. P-values were accordingly calculated to experiments with 50 μM Ca^{2+} (a dash in the P value columns). Asterisks indicate that statistical difference between appropriate ΔA_{540} values is not statistically significant.

Table S8 (Part 1)

Effects of EMA on RCR_{ADP} and RCR_{DNP} in succinate-energized rat liver mitochondria

0	5 mM Glutamate + 5 mM malate				5 mM Succinate				
**EMA** (μM)	**State 3**		**DNP-uncoupled state**		**EMA** (μM)	**State 3**		**DNP-uncoupled state**	
	RCR_{DNP} ± SEM	P value	RCR_{DNP} ± SEM	P value		RCR_{ADP} ± SEM	P value	RCR_{DNP} ± SEM	P value
0	1.97±0.09 (3)	-	2.46±0.07 (3)	-	0	2.45±0.13 (3)	-	3.84±0.27 (3)	-
50	1.04±0.02 (3)	P < 0.01	1.98±0.03 (3)	P < 0.01	5	2.22±0.12 (3)	*	4.22±0.13 (3)	*
					10	2.30±0.05 (3)	*	3.79±0.13 (3)	*
					30	1.80±0.12 (3)	P < 0.03	3.53±0.36 (3)	*
					50	1.74±0.10 (3)	P < 0.02	2.94±0.17 (3)	P < 0.05

Table S8 (Part 2)

Effects of FITC on RCR_{ADP} and RCR_{DNP} in succinate-energized rat liver mitochondria

0	5 mM Glutamate + 5 mM malate				5 mM Succinate				
**FITC** (μM)	**State 3**		**DNP-uncoupled state**		**FITC** (μM)	**State 3**		**DNP-uncoupled state**	
	RCR_{ADP} ± SEM	P value	RCR_{DNP} ± SEM	P value		RCR_{ADP} ± SEM	P value	RCR_{DNP} ± SEM	P value
0	1.88±0.13 (3)	-	2.34±0.11 (3)	-	0	2.31±0.02 (3)	-	3.56±0.09 (3)	-
100	1.25±0.03 (3)	P < 0.01	2.07±0.11 (3)	*	30	2.12±0.10 (3)	*	4.05±0.50 (3)	*
200	1.13±0.03 (3)	P < 0.01	1.81±0.12 (3)	P < 0.03	100	1.06±0.03 (3)	P < 0.01	1.97±0.30 (3)	P < 0.01
300	1.09±0.07 (3)	P < 0.01	1.48±0.07 (3)	P < 0.01	200	1.09±0.09 (3)	P < 0.01	1.31±0.03 (3)	P < 0.01
					300	1.01±0.01 (3)	P < 0.01	1.11±0.07 (3)	P < 0.01

Table S8 (Part 3)

Effects of TFP on RCR_{ADP} and RCR_{DNP} in succinate-energized rat liver mitochondria

0	5 mM Glutamate + 5 mM malate				5 mM Succinate				
	**State 3**		**DNP-uncoupled state**			**State 3**		**DNP-uncoupled state**	
**TFP** (μM)	RCR_{ADP} ± SEM	P value	RCR_{DNP} ± SEM	P value	**TFP** (μM)	RCR_{ADP} ± SEM	P value	RCR_{DNP} ± SEM	P value
0	1.88±0.13 (3)	-	2.34±0.11 (3)	-	0	2.30±0.03 (3)	-	3.66±0.12 (3)	-
50	1.43±0.10 (3)	P < 0.05	1.37±0.10 (3)	P < 0.01	50	1.51±0.05 (3)	P < 0.01	2.02±0.09 (3)	P < 0.01
100	1.15±0.12 (3)	P < 0.02	0.97±0.04 (3)	P < 0.01	100	1.01±0.01 (3)	P < 0.01	0.96±0.02 (3)	P < 0.01

Table S8 (Part 4)

Effects of Emb on RCR_{ADP} and RCR_{DNP} in succinate-energized rat liver mitochondria

0	5 mM Succinate			
	**State 3**		**DNP-uncoupled state**	
**Emb** (μM)	RCR_{ADP} ± SEM	P value	RCR_{DNP} ± SEM	P value
0	2.57±0.11 (3)	-	3.92±0.27 (3)	-
50	1.67±0.10 (3)	P < 0.01	2.37±0.39 (3)	P < 0.04
100	1.12±0.04 (3)	P < 0.01	1.30±0.11 (3)	P < 0.01

Values of RCR_{ADP} and RCR_{DNP} (see Fig. 8) are presented as Means ± SEM. The number of experiments showed in parentheses. P-values are calculated to experiments ftee additions of reagents (EMA, FITC, Emb, TFP). Asterisks indicate that statistical difference between appropriate values is not statistically significant.

Table S9

Effects of EMA, FITC, and $Cu(OP)_2$ on RCR_{DNP} in succinate-energized rat liver mitochondria

EMA			FITC			$Cu(OP)_2$		
**EMA** (μM)	RCR_{DNP} ± SEM	P value	**FITC** (μM)	RCR_{DNP} ± SEM	P value	**$Cu(OP)_2$** (μM)	RCR_{DNP} ± SEM	P value
0	2.16±0.07 (4)	-	0	2.16±0.07 (3)	-	0	2.05±0.17 (3)	-
10	1.76±0.05 (4)	P < 0.01	100	1.49±0.09 (3)	P < 0.01	3	1.70±0.06 (3)	*
30	1.67±0.07 (4)	P < 0.01	200	1.18±0.06 (3)	P < 0.01	3 + NEM	1.68±0.11 (3)	*
50	1.21±0.04 (4)	P < 0.01	300	0.52±0.08 (3)	P < 0.01			

Values of RCR_{ADP} and RCR_{DNP} (see Fig. 9) are presented as Means ± SEM. The number of experiments showed in parentheses. P-values are calculated to experiments ftee additions of reagents (EMA, FITC, $Cu(OP)_2$). Asterisks indicate that statistical difference between appropriate values is not statistically significant.

Table S10

Effects of EMA, FITC, $Cu(OP)_2$, and Cu^{2+} on RCR_{DNP} in succinate-energized and calcium-loaded rat liver mitochondria in the presence of MPTP inhibitors (ADP, CsA, NEM)

EMA				FITC			
	**EMA** (μM)	RCR_{DNP} ± SEM	P value		**FITC** (μM)	RCR_{DNP} ± SEM	P value
Ca^{2+}	0	0.59±0.05 (3)	-	Ca^{2+}	0	0.59±0.05 (3)	-
Ca^{2+}	10	0.95±0.05 (3)	P < 0.01	Ca^{2+}	100	0.60±0.06 (3)	*
Ca^{2+} + NEM	10	1.11±0.12 (3)	P < 0.02	Ca^{2+} + NEM	100	0.83±0.02 (3)	P < 0.01
Ca^{2+} + ADP + CsA	10	1.44±0.06 (3)	P < 0.01	Ca^{2+} + ADP + CsA	100	0.93±0.01 (3)	P < 0.01
Ca^{2+}	30	1.31±0.05 (3)	P < 0.01	Ca^{2+}	200	0.41±0.01 (3)	P < 0.02
Ca^{2+}	50	1.07±0.03 (3)	P < 0.01	Ca^{2+}	300	0.38±0.03 (3)	P < 0.02

**$Cu(OP)_2$**				**Cu^{2+}**			
	**$Cu(OP)_2$** (μM)	RCR_{DNP} ± SEM	P value		**Cu^{2+}** (μM)	RCR_{DNP} ± SEM	P value
Ca^{2+}	0	0.81±0.05 (3)	-		0	0.81±0.05 (3)	-
Ca^{2+}	3	1.53±0.04 (3)	$P < 0.01$		3	2.19±0.09 (3)	$P < 0.01$
Ca^{2+} + NEM	3	0.65±0.09 (3)	*	NEM	3	2.01±0.08 (3)	$P < 0.01$
Ca^{2+} + ADP + NEM	3	1.49±0.02 (3)	$P < 0.01$	Ca^{2+}	3	1.49±0.12 (3)	$P < 0.01$
Ca^{2+} + CsA + NEM	3	1.64±0.03 (3)	$P < 0.01$	Ca^{2+} + NEM	3	1.69±0.05 (3)	$P < 0.01$
Ca^{2+} + ADP + CsA + NEM	3	1.94±0.13 (3)	$P < 0.01$	Ca^{2+} + CsA + NEM	3	1.91±0.09 (3)	$P < 0.01$

**Control**			
		RCR_{DNP} ± SEM	P value
		2.34±0.05 (8)	$P < 0.01$
Ca^{2+}		0.68±0.06 (8)	-
Ca^{2+} + ADP + CsA		2.00±0.13 (7)	$P < 0.01$
Ca^{2+} + NEM		1.84±0.07 (7)	$P < 0.01$

Value of RCR_{DNP} (see Fig. 10) are presented as Means ± SEM. The number of experiments showed in parentheses. P-values are calculated to experiments with 100 μM Ca^{2+}. Asterisks indicate that statistical difference between appropriate values is not statistically significant.
